# Status and influencing factors of patients with kinesiophobia after insertion of peripherally inserted central catheter: A cross-sectional study

**DOI:** 10.1097/MD.0000000000029529

**Published:** 2022-07-29

**Authors:** Wang Liuyue, Gong Juxin, Huang Chunlan, Li Junli, Chen Liucui, Zhang Xialu, Liao Qiujiao, Liu Fangyin

**Affiliations:** a School of Nursing, Youjiang Medical University for Nationalities, Baise, China; b School of Clinical Medicine, Youjiang Medical University for Nationalities, Baise, China; c Department of Nursing, Affiliated Hospital of Youjiang Medical University for Nationalities, Baise, China; d Department of PICC Clinic, Affiliated Hospital of Youjiang Medical University for Nationalities, Baise, China.

**Keywords:** anxiety, influence factors, kinesiophobia, pain, peripherally inserted central catheter

## Abstract

This study aimed to investigate the current status and influencing factors of kinesiophobia in patients after insertion of peripherally inserted central catheter (PICC).

A total of 240 patients with PICC were included. Their postinsertion status and influencing factors were investigated using the general information questionnaire, Tampa Scale of Kinesiophobia (TSK), Medical Coping Modes Questionnaire, Numerical Rating Scale, and Self-rating Anxiety Scale.

The mean TSK score was 36.49 ± 4.19 points, and 89 patients (37.08%) had kinesiophobia. Multiple linear regression analysis showed that factors such as education level, age, monthly income level, catheterization history, face, pain level, anxiety, and number of needle insertions influenced postoperative kinesiophobia in patients with PICC (*P* < .05). The total variation in the TSK score was 71.8%.

The incidence of kinesiophobia was relatively high after PICC insertion. The medical staff needs to undertake targeted intervention measures to help minimize kinesiophobia after PICC insertion, allowing patients to perform scientifically correct functional exercises and attain physical recovery.

**Key points**

- The incidence of kinesiophobia was high after peripherally inserted central catheter (PICC) insertion; various influencing factors were education level, monthly income level, catheterization history, and pain degree.
- Nurses should educate and guide the patients on early ball-grip exercise for prognosis improvement and adopt a multidisciplinary cooperation approach among healthcare providers.
- The medical staff requires targeted intervention measures to help minimize kinesiophobia after PICC insertion that allows patients to perform scientifically correct functional exercises and attain physical recovery.
- Cognitive behavioral interventions are also necessary for effective pain management, thereby reducing kinesiophobia and improving patients’ rehabilitation exercise enthusiasm.

## 1. Introduction

Peripherally inserted central catheter (PICC) is a long, thin, flexible tube inserted through a peripheral vein.^[1]^ It can be indwelled for up to 1 year, and patients only need regular maintenance every week to keep it safe and reliable for a long time. PICC is especially suitable for patients who need long-term infusion or those with tumor requiring radiotherapy and chemotherapy; it is currently widely used clinically and is gradually recognized by patients.^[2,3]^ However, patients may experience pain and other discomforts after PICC insertion, causing fear of performing early ball-grip exercises and daily life and affecting the rehabilitation process.^[4]^ Kinesiophobia was first proposed according to the concept of the fear-movement-avoidance model. In 1999, Crombez et al^[5]^ reported that pain-related fear (fear of pain/physical activity/[re]injury) may be more disabling than the pain itself. Reneman et al^[6]^ later proposed that kinesiophobia refers to the individual’s’ excessive, unreasonable, and indiscreet fear of physical movement and activity, making them vulnerable to painful injury or reinjury. In kinesiophobia, the individual becomes particularly sensitive to pain and avoids engaging in sports or activities related to reinjury, leading to physical inactivity, thereby affecting the individual’s rehabilitation exercise and prognosis.^[7]^ Initially, kinesiophobia was more common in patients with chronic diseases, such as low back pain and heart disease, and later, it was widely used for evaluating surgical injuries and patients’ fear of exercise after surgery.^[8–11]^ This study aims to explore the current status and main influencing factors of kinesiophobia after PICC insertion in patients and to provide reference for intervention research.

## 2. Materials and Methods

### 2.1. Participants

By convenience sampling, we selected patients who were hospitalized in our hospital between July 2020 and December 2020 and who underwent ultrasound guidance combined with modified Sedinger technology PICC insertion as the research object.^[12]^ The catheter models are all Bard 4Fr polyurethane catheters with an open front end. The inclusion criteria were the following: consistent with PICC insertion indications; ≥16 years; upper limb as the PICC insertion site; and awareness and basic reading and communication skills. In contrast, the exclusion criteria were severe mental or language dysfunction and missing data. All patients in this study gave informed consent and reported to the ethics committee of our hospital for approval. A total of 240 patients aged 16 to 86 (51.51 ± 13.60) years completed the study. Regarding their education level, 112, 91, 19, and 18 were in elementary school and below, junior high school, high school and technical secondary school, and junior high school and above, respectively. Furthermore, 61, 106, 60, and 13 had a monthly income of ≤800, 801 to 2400, 2401 to 4000, and ≥4001 yuan, respectively.

### 2.2. Methods

#### 2.2.1. Research tools.

##### 2.2.1.1 General Information Survey Form.

In the general information survey form, the following were included: age, sex, nationality, education level, monthly income level, diagnosis, catheter model, catheter location (left/right hand or upper/lower elbow), catheter vein, tube insertion history, and number of needle insertions.

##### 2.2.1.2. Tampa Scale of Kinesiophobia.

The Tampa Scale of Kinesiophobia (TSK) is an outcome report scale designed to help patients identify motor phobia. It has a total of 17 items, and each item is scored between 1 point (strongly disagree) and 4 points (strongly agree). The total score of the scale is 17 to 68 points, with >37 points considered as panic disorder; the higher the score, the higher the patient’s level of panic disorder.^[13]^

##### 2.2.1.3. Numerical Rating Scale.

Numerical Rating Scale consists of 11 numbers (0–10), with 0 as “no pain” and 10 as “the most painful,” and the chosen number represents the level of pain.^[14]^

##### 2.2.1.4. Self-Rating Anxiety Scale.

Self-Rating Anxiety Scale is generally used to evaluate one’s anxiety level. It has 20 four-category items. The sum of the scores is the rough score, which is then multiplied by 1.25 to obtain an integer, which is then the standard score. The evaluation standard score of ≥50 indicates anxiety. In particular, 50 to 59, 60 to 69, and >69 are classified as mild, moderate, and severe anxiety, respectively.^[15]^

##### 2.2.1.5. Medical Coping Modes Questionnaire.

Containing 20 items, Medical Coping Modes Questionnaire is divided into 3 subscales: facing dimension (8 items), avoidance dimension (7 items), and yield dimension (5 items). The coefficients of these 3 subscales are 0.69, 0.76, and 0.60, respectively. Each item uses a 4-level classification method to calculate the total score of each dimension. The higher the total score, the more the patient is inclined to adopt this kind of response.^[16]^

#### 2.2.2. Data collection method.

With the assurance that the patient’s condition was stable after PICC insertion, we issued a questionnaire at the bedside, informed the patients of the purpose and precautions of the study, and asked them to fill out the questionnaire truthfully. If the patients could not fill it out by themselves, we would objectively describe the problem to them, and they would subsequently relay their answers to us. We would then check on the scale on behalf of the patient. General information was collected from the medical records, and usually, answering the questionnaire would take approximately 20 minutes. When recalling, we checked the questionnaire and returned it after filling up on the spot. Out of 258 questionnaires released, 240 valid cases were finally obtained, with an effectiveness rate of 93%.

#### 2.2.3. Statistical methods.

Data were processed using SPSS 22.0. Independent sample *t* test, 1-way analysis of variance, Pearson’s correlation analysis, and multiple linear regression analysis were also used. The test level was α = 0.05.

## 3. Results

### 3.1. Kinesiophobia, pain, medical coping style, and anxiety of patients after PICC insertion

After PICC insertion, the mean TSK score was 36.49 ± 4.19 points, and the incidence of kinesiophobia was 89 (37.08%). In the medical coping style, the face, avoidance, and yield scores were 19.23 ± 5.13, 15.14 ± 4.70, and 9.50 ± 3.13 points, respectively. Furthermore, the median pain score was 4 points, and the anxiety score was 54.64 ± 8.97 points.

### 3.2. Univariate analysis of patients’ TSK scores after PICC insertion

A univariate analysis of factors such as age, sex, education level, nationality, monthly income level, catheterization site, catheterization vein, catheterization history, number of needle insertions, and postoperative TSK scores were conducted (Table 1).

**Table 1:** Univariate analysis of patients’ kinesiophobia scores after PICC (n = 240).

**Categorical variables**	**Number of cases**	**TSK of scores**	**Statistical value**	***P* value**
Sex			*t* = 0.342	.733
Male	127	36.57 ± 4.24		
Female	113	36.39 ± 4.14		
Age			*F* = 11.582	.000
16–35	31	34.16 ± 4.27		
36–45	38	34.18 ± 3.49		
46–60	112	37.18 ± 4.27		
≥61	59	37.88 ± 3.30		
National			*F* = 0.018	.983
Han	40	36.60 ± 4.01		
Zhuang	190	36.46 ± 4.28		
Else	10	36.50 ± 3.24		
Education level			*F* = 2.778	.042
Primary school and below	112	37.19 ± 3.92		
Junior high school	91	36.23 ± 4.23		
High school and technical secondary school	19	35.05 ± 3.60		
College degree or above	18	34.94 ± 5.40		
Monthly income level			*F* = 9.303	.000
≤800	61	37.93 ± 4.07		
801–2400	106	36.99 ± 4.15		
2401–4000	60	34.75 ± 3.48		
≥4001	13	33.62 ± 4.33		
The vein of insertion			*F* = 1.406	.247
Basilic vein	201	36.48 ± 4.25		
Brachial vein	33	36.06 ± 3.24		
Median vein	6	39.17 ± 6.15		
Catheter site			*t* = 0.215	.830
Left side	73	36.58 ± 4.57		
Right side	167	36.45 ± 4.02		
The history of catheter			*F* = 11.364	.000
0	180	36.01 ± 3.74		
1	41	36.39 ± 4.54		
2	15	40.40 ± 4.75		
≥3	4	44.50 ± 2.65		
The needle number			*F* = 57.740	.000
1	178	35.10 ± 3.34		
2	36	39.72 ± 2.67		
≥3	26	41.50 ± 4.87		

### 3.3. Correlation analysis of kinesiophobia, coping style, anxiety, and pain in patients after PICC insertion

After PICC insertion, patients’ kinesiophobia negatively correlated with the face score (*r* = −0.773, *P* = .000) but positively correlated with the avoidance score (*r* = 0.833, *P* = .000), yield score (*r* = 0.589, *P* = .000), pain level (*r* = 0.545, *P* = .000), and anxiety (*r* = 0.623, *P* = .000).

### 3.4. Multiple linear regression analysis of patients with kinesiophobia after PICC insertion

Multiple linear regression analysis was performed. The patient’s TSK score after PICC insertion was the dependent variable, and the face score, anxiety score, pain score, and meaningful variables in the patient’s general information were the independent variables. We set the following values: α_in_ = 0.05 and α_out_ = 0.10. Factors such as the education level (primary school and below = 1, junior high school = 2, high school and technical secondary school = 3, and junior college and above = 4), per capita monthly income (≤800 yuan = 1; 801~2400 yuan = 2; 2401~4000 yuan = 3; and ≥4001 yuan = 4), face coping (original value input), pain (original value input), and anxiety (original value input) were included in the regression equation analysis. The total variation of the TSK score was 71.8% (Table 2). Concurrently, after the interaction term analysis of each variable in the model, no statistical difference was found; thus, it was determined that there was no interaction between variables that contributed to the kinesiophobia score.

**Table 2:** Multiple linear regression analysis of patients with kinesiophobia after PICC (n = 240).

**Variables**	**β**	**SE**	**β’**	***t***	***P* value**
Constant	40.616	1.945	–	20.883	.000
Age	0.608	0.176	0.137	3.450	.001
Education level	–0.458	0.179	–0.097	–2.555	.011
Monthly income level	–0.757	0.182	–0.153	–4.151	.000
The history of catheter	1.051	0.301	0.174	3.492	.001
The needle number	0.811	0.298	0.130	2.720	.007
Face	–0.445	0.041	–0.546	–10.745	.000
Pain	–0.236	0.104	–0.129	–2.276	.024
Anxiety	0.084	0.026	0.181	3.201	.002

## 4. Discussion

### 4.1. Kinesiophobia incidence after PICC insertion

In this study, the patient’s TSK score after PICC insertion was 36.49 ± 4.19 points, and the incidence of kinesiophobia was 37.08%, which was higher than that of patients after breast cancer surgery according to the Chinese scholar Sulan et al^[17]^ (29.59%). This discrepancy may be related to patients’ regional differences, cultural differences, and disease severity. This survey was conducted in Guangxi, where ethnic minorities gather, the old revolutionary base is relatively remote, and the economic development is relatively backward. Hence, 71.25% of our participants were over 45 years old, approximately 84.58% had studied till junior high school level, and 69.58% had a monthly income of <2400 yuan. Consequently, the incidence of kinesiophobia is higher in this study than in other studies. As mentioned, kinesiophobia refers to an individual’s excessive, unreasonable, and indiscreet fear of physical movement and activity. Individuals with kinesiophobia become particularly sensitive to pain and avoid participating in sports or activities related to reinjury, causing the body to be inactive. Body disuse affects individual rehabilitation function exercise and prognosis.^[18]^ Luque-Suarez et al^[19]^ showed that high levels of kinesiophobia are closely related to severe pain and disability. Thus, nurses should pay attention not only to the PICC line condition of patients but also to the patients’ cognitive behavior and psychological state to provide appropriate care.

### 4.2. Influencing factors of kinesiophobia after PICC insertion

#### 4.2.1. General information.

##### 4.2.1.1. Age.

This study showed that the older the PICC patients, the higher the TSK score. This result is consistent with that of the study by Cai et al. In this previous study, age, education level, and family monthly income per capita had statistically significant differences in kinesiophobia incidence in 298 patients with kinesiophobia after joint replacement.^[20]^ In addition, the older the patients, the more they became sensitive to pain, the slower they could accept new technologies (limited use of smartphones for WeChat, Weibo, and browsers), and the more they insisted on believing certain traditional misconceptions. Generally, patients are relatively unfamiliar with PICC placement (a new technique for intravenous therapy), causing fear and agitation. Therefore, nurses should pay attention to the corresponding PICC postinsertion panic care measures for people of different ages.

##### 4.2.1.2. Education level.

This study found that in patients with PICC, those with a high level of education had mild kinesiophobia, consistent with the finding of Gunay Ucurum^[21]^, who believes that as the level of education decreases, the score of kinesiophobia increases. Patients with a high level of education have more abilities and resources to improve their disease-related and PICC insertion knowledge, to gain correct cognition, to be compliant, and to be able to rationally treat PICC insertion for a long time. The higher the knowledge level, the lower the degree of kinesiophobia.^[22]^ Conversely, patients with PICC with low levels of education would think that PICC has foreign body sensation and is inconvenient to move. They would dare not to move and be overly worried about the risk of prolapse, blockage, or thrombosis in the pipeline. Hence, in health education, the medical staff should focus on the use of multimedia to correct the patients’ understanding of exercise, pain, and catheterization according to the different education levels and comprehension capabilities of patients.^[23,24]^ They should also cooperate with the patients in developing practical and feasible postinsertion functional exercise programs that meet patients’ expectations to increase their voluntary participation, overcome their fear of PICC exercise, and reduce the rate of reinsertion and the incidence of complications such as thrombus and blockage.^[25]^

##### 4.2.1.3. Income level.

The level of kinesiophobia in patients with PICC negatively correlated with the income level, consistent with the findings of Ioannou et al^[26]^ through a survey of 433 patients in the orthopedics department of a large trauma hospital in Melbourne. The reason could be that the PICC needs to be carried for a long time after its placement, demanding a certain amount of time and money to maintain the pipeline in a qualified hospital on time. Especially for patients living in remote areas, financial worries and recovery prognosis are accompanied with huge injuries, and they experience continuous sickness, leading to poor physical health and high psychological distress. Moreover, most of the patients are older; hence, they have a long recovery period and needs to be taken care of. After PICC insertion, they cannot engage in heavy physical labor, causing fear of greater financial pressure on themselves and even their families. Cai et al^[27]^ analyzed the incidence and risk factors of kinesiophobia after total knee arthroplasty in 862 patients in Zhengzhou; they found that lower income levels were related to kinesiophobia probability after surgery. Therefore, the medical staff needs to help patients with PICC fully grasp the method of functional exercise after catheterization, and inform them that effective and appropriate rehabilitation training can help protect the vein, protect the indwelling PICC, avoid complications and secondary punctures, and help relieve patients from economic pressure to restore health as soon as possible.^[28]^

##### 4.2.1.4. History of PICC placement and number of needle insertions.

This study demonstrated that the higher the number of catheterizations and the number of needle insertions, the higher the degree of kinesiophobia. The first time the tube is inserted or the tube is successfully delivered with a single needle, the process is smoother, the patient suffers less pain, and the fear of tube insertion is reduced, thereby improving patient’s confidence in the treatment after PICC insertion.^[29]^ However, when the catheter is inserted multiple times or the number of needle insertions is high, the pain level is high, the catheterization becomes more expensive, and the PICC may fail again or cannot be successfully retained for a long time. Consequently, they will distrust the ability of the catheterization personnel and doubt the state of their own blood vessels. Patients’ fear of PICC placement and early postinsertion activities will increase significantly; thus, they will be hesitant on participating in early ball-grip training, making themselves prone to postoperative complications. Therefore, the medical personnel are encouraged to conduct health education before and after PICC, lessen misunderstandings, and improve patient confidence. Especially, they should provide a complete and appropriate health instruction before hospital discharge, instruct the patients to have regular follow-ups, and improve patient satisfaction with nursing services. At the same time, they should continue to improve their business capabilities, strengthen the technical level of PICC placement, and increase the success rate of puncture without increasing patient pain and burden.^[30,31]^

#### 4.2.2. Face-to-face response.

The more the patients tended to face their fear, the lower the degree of kinesiophobia. In contrast, the more they tended to avoid and yield to coping, the higher the degree of panic disorder. Yuhua et al^[32]^ found in their study of 600 patients with osteoporotic kinesiophobia that the more inclined they were to deal with it, the more confident they were to overcome their fear of pain and fear of movement and the more active they are in participating in functional exercises and have a strong sense of self-efficacy. Positive coping can make patients fully confident in PICC intravenous treatment and treatment of related diseases. When facing problems, positive patients will take the initiative to seek social support from family, friends, and the medical staff. Therefore, the medical staff should also focus their care on patients who have the correct active coping behavior in dealing with fear of PICC insertion but do not adapt.^[33]^ They should also timely identify patients who avoid and yield a negative response. In addition, family members should actively encourage their patients mentally and emotionally to fully support them, help them overcome their fear, and get back to health.

#### 4.2.3. Degree of pain.

Patients with a higher degree of pain had a higher level of kinesiophobia after PICC insertion. PICC placement can effectively support cancer chemotherapy and protect patients’ veins, but patients experience pain from ball-grip exercise and puncture. Patients are often afraid of activities and resist early exercise because of continuous pain. Pain experience often causes individuals to have fear and kinesiophobia beliefs, resulting in physical inactivity to reduce the injury or pain caused by exercise.^[34]^ However, the avoidance behavior generated by fear avoidance belief cannot relieve the pain; instead, it leads to limited daily activities and reduced life and work ability of the patient, the state of “disease waste,” and the possibility of thrombosis and other complications.^[35,36]^ Therefore, conducting active pain management through multidisciplinary cooperation after PICC insertion is important. Meanwhile, patients should be educated on pain to correctly recognize the relationship between pain and activities and enlighten them that early ball-grip exercise is essential to achieve long-term retention of PICC insertion.^[37]^

#### 4.2.4. Psychological factors.

The anxiety symptoms of patients with PICC insertion positively correlated with the kinesiophobia level. Pells et al^[38]^ found that higher levels of exercise phobia were associated with greater psychological distress, especially anxiety, psychosis, obsessive compulsive disorder, depression, and interpersonal sensitivity. Patients with PICC and kinesiophobia itself will bear a heavy psychological burden, causing anxiety, depression, and other negative emotions,^[39]^ plus In addition, they experience pain in early ball-grip exercise and need to live with a tube for a long time with treatment, thereby aggravating anxiety. Anxiety, depression, and other psychological factors caused by kinesiophobia will affect the recovery effect of the disease.^[40]^ Therefore, medical personnel should advocate on patient-centered approach.^[40]^ Nurses should pay attention not only to the physical aspect of the disease but also to the emotional and mental state of the patients. Relieving patients’ anxiety also needs multidisciplinary collaboration among healthcare providers, including psychologists (Kang Fushi), whom patients can express their emotions with.

#### 4.2.5. Other factors.

In addition to physical, psychological, and social factors, we should also consider other related factors, such as whether the side of catheterization is the patient’s preferred side, which may also affect the patient’s dyskinesia score. If the patient uses the right hand to perform activities and the right arm is also used for catheterization, then the patient may feel that a PICC affects normal activities, increasing the fear of movement and discomfort. Therefore, the medical staff should fully understand the daily habits of patients before catheterization and try to avoid puncturing the dominant hand.

## 5. Conclusion

The incidence of kinesiophobia was high after PICC insertion, and among the influencing factors were education level, monthly income level, catheterization history, and pain degree. Nurses should improve the backwardness of patients with PICC to correct kinesiophobia misconceptions. They should also educate and guide the patients on early ball-grip exercise for prognosis improvement and adopt a multidisciplinary cooperation approach among healthcare providers. Cognitive behavioral interventions are also necessary for effective pain management, thereby reducing kinesiophobia and improving patients’ rehabilitation exercise enthusiasm. The limitations of this study include the relatively small overall sample size and the heterogeneity between the groups; thus, a higher number of selected samples and a wider scope in future research is necessary to make the research results more meaningful.

## Author contributions

Conceptualization: Liu Fangyin, Huang Chunlan.

Data curation: Wang Liuyue, Gong Juxin, Li Junli.

Formal analysis: Wang Liuyue, Chen Liucui, Zhang Xialu, Liao Qiujiao.

Investigation: Wang Liuyue, Li Junli, Chen Liucui, Zhang Xialu, Liao Qiujiao.

Methodology: Wang Liuyue, Liu Fangyin.

Project administration: Wang Liuyue.

Resources: Liu Fangyin, Huang Chunlan, Li Junli.

Software: Wang Liuyue, Liu Fangyin, Zhang Xialu.

Supervision: Wang Liuyue, Liu Fangyin.

Writing-original draft: Wang Liuyue, Gong Juxin, Liu Fangyin.

Writing-review & editing: Wang Liuyue, Liu Fangyin, Huang Chunlan.
